# Glucagon secretion from pancreatic α-cells

**DOI:** 10.3109/03009734.2016.1156789

**Published:** 2016-03-24

**Authors:** Linford Briant, Albert Salehi, Elisa Vergari, Quan Zhang, Patrik Rorsman

**Affiliations:** 1 Oxford Centre for Diabetes, Endocrinology and Metabolism, Radcliffe Department of Medicine, University of Oxford, Oxford, UK;; 2 Metabolic Research, Department of Physiology, Institute of Neuroscience and Physiology, University of Göteborg, Göteborg, Sweden

**Keywords:** diabetes, electrophysiology, experimental diabetes, glucagon, intrinsic mechanisms, pancreatic alpha-cells, paracrine

## Abstract

Type 2 diabetes involves a ménage à trois of impaired glucose regulation of pancreatic hormone release: in addition to impaired glucose-induced insulin secretion, the release of the hyperglycaemic hormone glucagon becomes dysregulated; these last-mentioned defects exacerbate the metabolic consequences of hypoinsulinaemia and are compounded further by hypersecretion of somatostatin (which inhibits both insulin and glucagon secretion). Glucagon secretion has been proposed to be regulated by either intrinsic or paracrine mechanisms, but their relative significance and the conditions under which they operate are debated. Importantly, the paracrine and intrinsic modes of regulation are not mutually exclusive; they could operate in parallel to control glucagon secretion. Here we have applied mathematical modelling of α-cell electrical activity as a novel means of dissecting the processes that underlie metabolic regulation of glucagon secretion. Our analyses indicate that basal hypersecretion of somatostatin and/or increased activity of somatostatin receptors may explain the loss of adequate counter-regulation under hypoglycaemic conditions, as well as the physiologically inappropriate stimulation of glucagon secretion during hyperglycaemia seen in diabetic patients. We therefore advocate studying the interaction of the paracrine and intrinsic mechanisms; unifying these processes may give a more complete picture of the regulation of glucagon secretion from α-cells than studying the individual parts.

## Introduction

When Banting and Best (1) injected extracts from pancreatic tissue intravenously or subcutaneously into diabetic dogs and humans, they observed a marked reduction in blood sugar (reflecting the hypoglycaemic action of insulin). However, this was often preceded by a small transient hyperglycaemia, which was initially attributed to epinephrine. A few years later, this phenomenon was suggested to be due to a glucose-mobilizing substance, later named ‘glucagon’ (2). It was another 30 years before the pancreatic α-cells were identified as being the source of glucagon (3), with hypoglycaemia demonstrated as triggering the release of this hormone (4,5).

Glucagon is part of a homeostatic hormonal system developed to protect against serious decreases in blood glucose—glucose ‘counter-regulation’. This mechanism is the combination of processes that act to protect against the development of hypoglycaemia and (should this occur) restore normoglycaemia. Hypoglycaemia suppresses insulin secretion from β-cells and stimulates glucagon secretion from islet α-cells, normalizing blood glucose levels. Even small changes in glucagon can greatly increase blood glucose; the addition of minimal doses of glucagon (0.50 ng/kg/min) is known to induce long-lasting hyperglycaemia (6). Glucagon acts exclusively on the liver, where it stimulates both glycogenolysis (the breakdown of glycogen into glucose) and gluconeogenesis (the formation of new glucose molecules), increasing glucose output within minutes. Under certain conditions, glucagon can also stimulate production of ketone bodies in the liver, which during fasting or prolonged hypoglycaemia may substitute partially for glucose in meeting the brain’s energy needs.

Hormones secreted from pancreatic islet cells play central roles in the whole-body glucose homeostasis. The dysfunction of these endocrine cells contributes to both type 1 and type 2 diabetes mellitus (7), with type 2 diabetes mellitus (T2DM) accounting for 90% of the total diabetes incidence (8). T2DM is a major and increasing health problem (9) with a complex aetiology. Both genetic and environmental factors are known to be involved in the disease (10). There is an excessive production of glucose by the liver, which contributes to the fasting hyperglycaemia that is characteristic of this disease. This pathological response is commonly attributed to a combination of insulin resistance and a failure of pancreatic β-cells to release insulin as required (11). However, it is becoming increasingly apparent that impaired glucagon secretion also contributes to the excessive hepatic glucose production in T2DM, exacerbating episodes of hyperglycaemia (12–14). Impaired secretion of glucagon in T2DM shows itself in a two-fold manner: as well as there being too much glucagon secretion during hyperglycaemia, there is also too little glucagon released to normalize hypoglycaemia (15). This compromised physiological and behavioural response to falling blood glucose concentrations is arguably the more fatal arm of impaired glycaemic control in T2DM, with hypoglycaemia being a major cause of mortality in insulin-treated patients (16).

Whereas the mechanisms by which glucose regulates insulin secretion from the β-cells have been well established (17–19), factors regulating glucagon secretion from α-cells in response to glucose belong to the most contested aspects of islet cell biology (20–22). The heart of the question is whether glucagon secretion is regulated by an intrinsic mechanism (within the α-cells) or by factors released from other cells within the islets (paracrine mechanisms). In this review, we summarize the intrinsic and paracrine mechanisms contributing to the glucose control of glucagon secretion in pancreatic α-cells. We also attempt to combine the intrinsic and paracrine mechanisms using mathematical models to address the possible cause of impaired glucagon secretion seen in diabetes.

## Paracrine regulation of glucagon secretion

Since glucose stimulates insulin release, and insulin inhibits glucagon secretion, it is inviting to think that inhibition of glucagon secretion from α-cells is secondary to stimulation of the β-cells. A similar argument could be made for somatostatin, the release of which is also stimulated by glucose. The argument that paracrine factors influence glucagon secretion is supported by reports that isolated α-cells (that no longer have paracrine input) are unable to respond appropriately (i.e. decreasing their activity and glucagon secretion) to increased glucose concentrations; in fact, the reports demonstrate that glucose *stimulates* glucagon secretion when α-cells are removed from their normal paracrine environment (23,24). Insulin was the first paracrine factor from β-cells to provide evidence for this inhibitory action (25). GABA is also released from β-cells (26), and some studies have demonstrated it can inhibit glucagon secretion from α-cells by activation of the GABA(A) receptor (27). Zn^2+^ (co-released with insulin) has been suggested to have an important role in glucagon secretion (28,29), but this has been questioned (30). Indeed, in mice where the Zn^2+^ granule transporter is knocked out, there was no alteration in the regulation of glucagon secretion by glucose (31).

Other studies refute the centrality of the control of glucagon secretion by insulin; they demonstrate that single (isolated) α-cells do respond to high glucose by lowering [Ca^2+^]_i_ and decreasing glucagon secretion (32,33). Furthermore, in 5 mM glucose, glucagon secretion is maximally inhibited in mouse islets—a concentration not associated with any change in insulin secretion (22,34). In fact, insulin actually *stimulated* glucagon secretion in islets exposed to 6 mM glucose. This may explain the paradoxical stimulation of glucagon secretion that occurs in line with increasing insulin secretion (for glucose concentrations ≥6 mM in mouse islets). Therefore, the inhibition of glucagon secretion in <6 mM glucose is not due to insulin secretion.

Somatostatin (SST) is a potent inhibitor of insulin and glucagon secretion. It has been proposed to be a paracrine regulator of glucagon secretion (35) with an important role for inhibiting glucagon secretion during hyperglycaemia (36). α-Cells in islets express somatostatin receptor 2 (SSTR2) (37). Glucagon secretion is increased in islets in which the SSTR2 is knocked out, highlighting SST as a mediator of the glucose inhibition of glucagon secretion (38).

SST exerts its inhibitory effect at the level of membrane potential and cell exocytosis. Upon binding to the SSTR2, SST activates a G-protein coupled inwardly rectifying K^+^ channel (GIRK), which repolarizes the cell membrane and inhibits the firing of action potentials (39). This effect in membrane potential is transient, probably due to the desensitization of the receptors. SST also inhibits α-cell exocytosis by effectively decreasing the intracellular cAMP level (37,40). With increasing glucose concentrations in the range 0–7 mM, SST release increases in parallel with the decrease in glucagon secretion (22,34). Therefore, it may be argued that inhibition of glucagon secretion is caused by SST signalling. However, glucose retains an inhibitory influence on glucagon secretion in the presence of CYN154806, an SSTR2-specific blocker (34), suggesting that SST does not regulate glucagon secretion (41). Furthermore, a number of different laboratories have demonstrated that blocking the SST signalling pathway in α-cells—either by blocking SST receptors (42) or the associated G-protein cascade (43)—increases glucagon secretion but does not prevent inhibition of glucagon release by glucose. In particular, SST does inhibit glucagon secretion at 0–7 mM glucose, but there is an SST-independent pathway that inhibits glucagon secretion in α-cells.

Taken together, these studies indicate that glucose exerts regulation of glucagon secretion independently of the paracrine factors, via intrinsic mechanisms. Interestingly, the ability of the incretin hormone GLP-1 to inhibit glucagon secretion in the perfused rat pancreas was abolished after immunoneutralization of somatostatin, whilst the effect of glucose was unaffected (44), indicating that the significance of paracrine and intrinsic mechanisms in the control of glucagon secretion is variable.

## Intrinsic regulation of glucagon secretion

Pancreatic α-cells, like β-cells, are electrically excitable. At low concentrations of glucose, when the secretion of glucagon is stimulated, α-cells fire continuous overshooting action potentials (APs) (23,27,45). Voltage-gated Na^+^ channels (Na_v_ channels) and Ca^2+^ channels contribute to the upstroke of APs (46,47). The discharge of high-voltage APs opens voltage-gated Ca^2+^ channels (VGCCs) to allow influx of extracellular Ca^2+^ into the cytosol. This results in an elevation of the intracellular Ca^2+^ concentration and provides Ca^2+^ signals that trigger glucagon granule exocytosis—a process that makes hormone-containing granules fuse to the cell membrane to release their cargo. Pharmacological studies have confirmed that there are at least four different VGCCs (T, L, N, and P/Q-type Ca^2+^ channels) expressed in the α-cells (46,48,49). Among them, the P/Q-type Ca^2+^ channel has been identified as the main VGCC that is involved in glucagon secretion evoked by low glucose despite its relatively low contribution in the transmembrane Ca^2+^ current (∼30%) (46). The L-type Ca^2+^ channel, although contributing the majority of Ca^2+^ currents (>50%), plays a minor role in the glucagon secretion under these conditions (50,51). This suggests that there is a tight coupling of the P/Q-type Ca^2+^ channels and the exocytotic machinery of the α-cells—which is likely to be compartmentalized. Such arrangement avoids the spill-over of Ca^2+^ from other VGCCs and enables glucagon secretion be regulated by modulation of P/Q-type Ca^2+^ channel activity.

**Figure 1.**
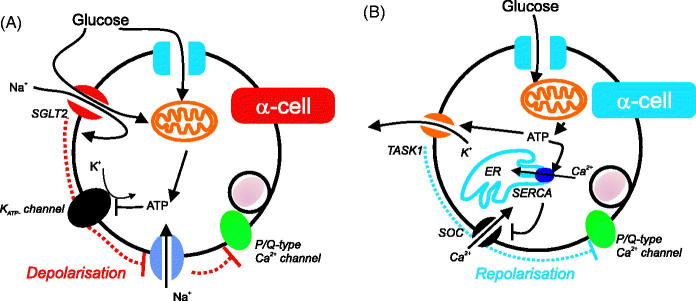
The glucose control of glucagon secretion from α-cells via intrinsic mechanisms. (A) K_ATP_ channels and SGLT2 depolarize the cell, decreasing action potential height and therefore P/Q activity. This results in reduced glucagon secretion. (B) TASK1 and store-operated channels (SOC) have been proposed to increase repolarizations in the cell, decreasing action potential frequency, and therefore glucagon secretion.

When the circulating glucose level rises, glucagon secretion is suppressed. This is likely to be via the reduction of P/Q-type Ca^2+^ channel activity in α-cells. Such inhibitory effect can be achieved by lowering the amplitude or the firing frequency of APs, by influencing membrane depolarization or repolarization, respectively (Figure 1). The change of membrane potential is a result of glucose metabolism or transport (via electrogenic sodium-glucose co-transporter 2 transporters (52) as discussed below), which leads to the alteration of membrane ion conductance. α-Cells are equipped with ATP-sensitive K^+^ channels (K_ATP_ channels) of the same molecular identity as in β-cells (46,48,51). Increasing glucose concentrations result in increased glucose metabolism and ATP production, inhibiting the K_ATP_ channel. This in turn leads to membrane depolarization (Figure 1A). Consequently, the amplitude of APs reduces due to voltage-dependent inactivation of Na_v_ channels. As a result, APs, although still being generated, cannot reach the voltage that is sufficient to open P/Q-type Ca^2+^ channels. The result is that secretion of glucagon is reduced.

Besides restriction of K^+^ conductance, membrane depolarization can also be induced by influx of positively charged ions. In α-cells, this can occur at the stage of glucose transport. In addition to glucose transporters (GLUTs), α-cells, in particular in human islets, also express sodium-glucose co-transporter 2 (SGLT2) (52). When glucose concentrations are high, SGLT2 transports glucose into the α-cell together with Na^+^ with a ratio of 1:1 (53). Thus, positive charges can be introduced into cells before glucose undergoes metabolism, which will lead to membrane depolarization. Although there are no direct electrophysiological data about the role of SGLT2 in α-cell membrane potential, it has been reported that blocking it with dapagliflozin could lift the inhibitory effect of glucose on glucagon secretion (52).

The central role of K_ATP_ channels and SGLT2 in the suppression of glucagon secretion from α-cells by reducing action potential height (through membrane depolarization) is contested. There are reports that suggest that glucagon secretion is controlled in a different manner: glucose metabolism increases repolarization of the α-cell membrane (reducing its firing frequency, and therefore secretion of glucagon) by either reducing depolarizing cation conductances or enhancing repolarizing K^+^ conductances (Figure 1B). It has been proposed that α-cell excitability at low glucose concentrations was maintained by a store-operated Ca^2+^-channel (SOC) (21,22,42,54). In high glucose, ATP generated from glucose metabolism promotes Ca^2+^ sequestration into the endoplasmic reticulum (ER) by activating sarco/endoplasmic reticulum Ca^2+^-ATPase (SERCA). Consequently, the SOC is closed and the cell membrane repolarizes. In addition, the increasing ATP level due to high glucose can also activate a two-pore domain K^+^ channel—TWIK-related acid-sensitive K^+^ channel 1 (TASK1 channel), thus providing repolarizing K^+^ conductance (55). Both mechanisms suggest that a rise of glucose concentration/metabolism leads to the inhibition of intracellular Ca^2+^ oscillation and thus ceased secretion. These studies were conducted using dispersed single cells that went through mechanical and/or enzymatic dispersing processes. However, most studies on intact islets indicate that the α-cell Ca^2+^ oscillations persist at high (inhibitory) glucose concentrations (46,56). These divergent results may therefore be a result of isolated/cultured α-cells being different from α-cells *in situ* in terms of their electrophysiology. For example, Na^+^ currents are reduced 10-fold in dispersed single α-cells (57) compared to those recorded from intact islets (46).

## Combining intrinsic and paracrine mechanisms to understand glucagon secretion

**Figure 2.**
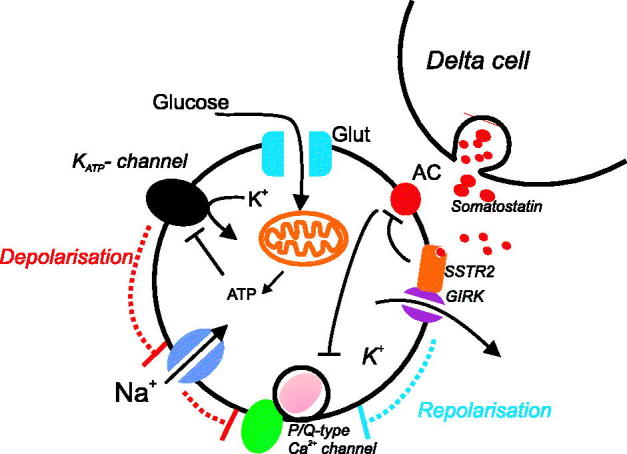
Combining intrinsic and paracrine regulation of glucagon secretion. See main text for details.

**Figure 3.**
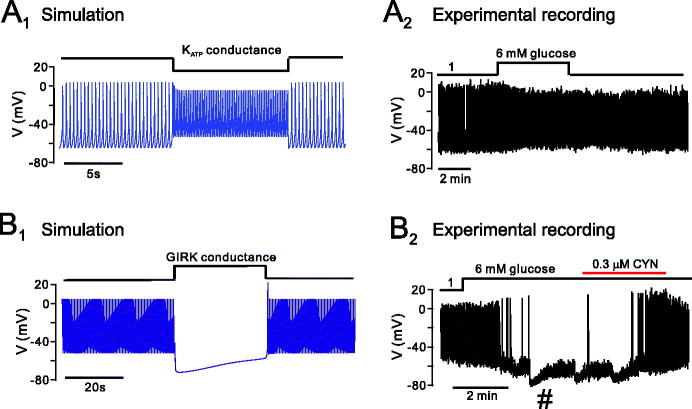
Mathematical modelling of the involvement of K_ATP_ and GIRK channels in the response of α-cells to high glucose. The mathematical model was simulated under conditions of a stepwise increase in K_ATP_ (A) and GIRK channel (B) activity, to mimic the action of high glucose on α-cell activity through intrinsic (K_ATP_) and paracrine (GIRK) activity. When conductance of the K_ATP_ channel was decreased in the model (A_1_), action potential frequency increased but amplitude decreased, as can be seen experimentally when α-cells are exposed to high glucose (A_2_). B: In some α-cells, exposure to high glucose causes spontaneous repolarizations (**#**) that are blocked by the SST receptor antagonist CYN (and are therefore thought to be due to periodic SST release; see (46)). We therefore mimicked this by increasing the GIRK conductance in the model (B_1_). The model was able to produce spontaneous repolarizations, as seen experimentally (B_2_). K_ATP_ = ATP-sensitive potassium channel; GIRK = G-protein coupled inwardly rectifying channel; SST = somatostatin.

It is important to emphasize that these intrinsic/paracrine mechanisms are not mutually exclusive. However, major uncertainty remains about their relative roles and whether they interact. For example, at a glucose level where glucagon is maximally inhibited (5–7 mM), although little insulin is secreted, somatostatin secretion is already being stimulated (34,42). Thus it may be that both the aforementioned intrinsic and paracrine effects of SST govern the glucose control of glucagon secretion in low glucose (Figure 2). It is interesting to notice that, in the presence of SSTR2-specific blockers, even at a very low concentration of glucose (below the stimulatory level for somatostatin secretion), glucagon secretion is much higher than that measured under control conditions (42,46). Such an observation suggests that glucagon secretion is under tonic inhibition by somatostatin. Indeed, we have observed that 60% (16 out of 27) of α-cells exhibited transient spontaneous repolarizations (possibly due to the activation of GIRK channels by SST; see Figure 3B2 for an example) when sensing 1 mM glucose. The occurrence of such repolarizations only moderately increased (65%; 9 out of 14 cells) when a high concentration of glucose was applied. We have found that although δ-cells are stimulated by glucose, some (<10%) δ-cells are spontaneously active already at low (1 mM) glucose (E. Vergari and P. Rorsman, unpublished observations).

Unifying these intrinsic and paracrine mechanisms could provide a more complete picture of the regulation of glucagon secretion in health and disease. A pharmacological and electrophysiological dissection of how these paracrine and intrinsic mechanisms interact to regulate glucagon secretion would be technically challenging. Therefore we resorted to mathematical modelling, a powerful complement to understanding biological systems (58), which provides a useful tool for investigating complex dynamic systems.

Hodgkin–Huxley-like formalisms of the membrane ion channels in α-cells allow the modelling of the α-cell membrane potential. A number of such mathematical models of α-cells have been created and used to study the mechanisms regulating glucagon secretion (59–62).

We investigated whether K_ATP_ and GIRK channel activity could explain glucose counter-regulation. We generated a model of the α-cell membrane potential, described by the following differential equation:
(Eq. 1)
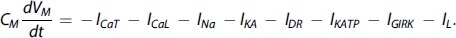


Here we have assumed that the membrane potential dynamics are governed by T-type and L-type calcium currents (*l_CaT_* and *l_CaL_*), a fast-activating Na^+^ current (*l_Na_*), potassium currents of the delayed rectifying-type (*l_DR_*) and A-type (*l_A_*), and a passive leak current (*l_L_*). We also have currents due to a G-protein coupled inwardly rectifying (*l_GIRK_*) and ATP-sensitive potassium channel (*l_KATP_*). All equations for these currents are as in Watts and Sherman (59), except *l_GIRK_* which is given by:
(Eq. 2)
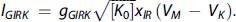


Here, *g_GIRK_* is the maximal conductance density of the GIRK channel (governed by activation of SSTR2), *x_lR_* is a gating variable, *V_K_* is the potassium reversal potential, and [*K_o_*] is the extracellular potassium concentration (see Fink et al. (63)). We mimic changes in GIRK and K_ATP_ channel activity (driven by changes in glucose concentration) by modulating the maximal conductance densities of the K_ATP_ and GIRK channel, *g_KATP_* and *g_GIRK_*, respectively (Figure 3). We find that decreasing *g_KATP_* depolarizes the membrane potential and reduces action potential height, as seen experimentally when α-cells are exposed to high glucose (Figure 3A1_,2_). When we force the model with *g_GIRK_* (to mimic periodic SST release), we see that the model exhibits a spontaneous repolarization, as seen experimentally in a subpopulation of α-cells (that are proximal to δ-cells) in response to high glucose (Figure 3B1_,2_). The model can therefore explain the different membrane potential responses to high glucose in α-cells.

## Glucose control of glucagon release and T2DM

As previously mentioned, insufficient insulin secretion and insulin resistance are exacerbated in T2DM by too much glucagon secretion during hyperglycaemia and too little during hypoglycaemia (64). However, the contribution of K_ATP_ channels to impaired regulation of glucagon secretion in T2DM is unclear. It is interesting to note that the inversion of glucagon secretion seen in T2DM can be mimicked in a mouse model with deficient K_ATP_ channels (Kir6.2STOP (34)). However, this mouse model revealed that high-glucose stimulation of glucagon secretion must have a K_ATP_-channel-independent mechanism.

**Figure 4.**
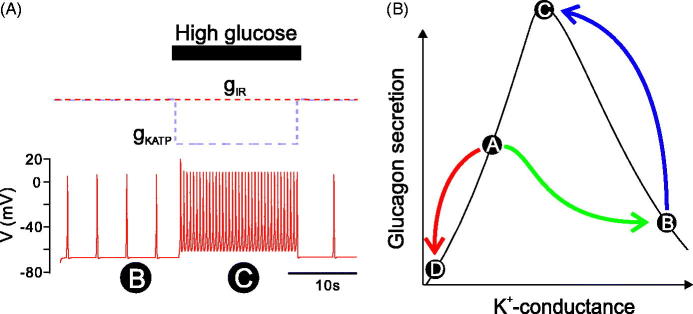
The interaction of intrinsic and paracrine mechanisms in the regulation of glucagon secretion in T2DM. (A) The mathematical model was simulated under conditions of increased GIRK conductance, to mimic the increased SST seen in diabetes. Under low glucose, the model fired with low-firing frequency (associated with low secretion of glucagon). When the K_ATP_ conductance was reduced (to simulated high glucose), the cell depolarized and firing frequency increased. However, there was no significant increase in action potential height. Therefore, glucagon secretion would be expected to increase in response to high glucose. This model therefore demonstrates the inverted regulation of glucagon secretion seen in diabetes. (B) In a healthy cell in low glucose, K_ATP_ activity is high and GIRK activity is nearly minimal 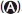. Glucagon secretion is high. If the healthy cell is exposed to high glucose, the K_ATP_ channel closes, causing glucagon secretion to reduce 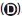. In T2DM, SST secretion in low glucose is increased (E. Vergari & P. Rorsman, manuscript in preparation). This pushes the α-cell over the bell into a state of low secretion of glucagon 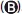. High glucose, which closes K_ATP_ channels, will now reduce the total K^+^ conductance, pushing the cell through the peak of the bell shape, increasing glucagon secretion 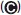. T2DM = type 2 diabetes mellitus; K_ATP_ = ATP-sensitive potassium channel; GIRK = G-protein coupled inwardly rectifying channel; SST = somatostatin.

It is of great interest that SST receptor antagonists can correct the defective counter-regulation of glucagon secretion observed in diabetic rats (65,66). This is consistent with reports that in diabetes there is an increase in the number of δ-cells (67–70). The resultant hypersecretion of SST may, via binding to SSTR2 and activation of GIRK channels, suppress glucagon secretion. However, this hypothesis remains to be experimentally validated. To mimic this increase in SST release in diabetes, we increased *g_GIRK_* in the mathematical model (increased SST release would cause hyperactivation of GIRK channels). We then mimicked exposure to high glucose by decreasing K_ATP_, and the model demonstrated an increase in firing frequency, but no change in action potential firing (Figure 4A). Hence the secretion of glucagon in high glucose would be expected to increase in this situation; the model therefore demonstrates that a combination of overactivity of GIRK and the depolarization due to K_ATP_ closure may explain the loss of counter-regulation of glucagon secretion seen in T2DM.

Based on these simulation data we therefore propose a mechanism by which impaired counter-regulation of glucagon secretion is generated in T2DM (Figure 4B): In healthy α-cells, GIRK channel activity at low glucose is relatively low (due to low basal SST release) and K_ATP_ activity remains sufficiently high to prevent Na_v_ inactivation. As a result, the α-cell fires large-amplitude action potentials, and glucagon secretion is high. When glucose is elevated, K_ATP_ channel activity becomes completely inhibited and the resultant membrane depolarization (via inactivation of Na_v_ and reduced action potential amplitude) leads to suppression of glucagon secretion.

In T2DM α-cells, there is a higher K^+^ conductance due to increased K_ATP_ channel activity (46) and hyperactivation of GIRK channels as the consequence of SST over-secretion (as suggested by the ability of SST antagonists to increase glucagon (38,39)). The high resting K^+^ conductance moves the membrane potential to a state of reduced action potential firing. When such T2DM α-cells are exposed to high glucose, glucose leads to the closure of K_ATP_ channels but GIRK channel activity is unaffected, and in these cells the response to glucose is similar to what is seen in β-cells: action potential firing is increased due to the membrane depolarization, but the depolarization is not strong enough to cause a reduction of the action potential height. Therefore, in contrast to the non-diabetic α-cells, increasing glucose results in a *stimulation* of secretion of glucagon in T2DM. These intrinsic and paracrine mechanisms can, when taken together, explain the impaired regulation of glucagon secretion seen in T2DM.

## Conclusions

We have attempted to illustrate the power of combining experimental data with mathematical modelling. It demonstrated, within certain ranges of glucose concentrations, that both intrinsic and paracrine mechanisms can operate in parallel to regulate glucagon secretion from the pancreatic islet α-cells. In particular, the analyses underscore the important physiological role played by intra-islet somatostatin signalling. It should be pointed out that, in this study, we have restricted our analysis to the electrical activity. However, in addition to its ability to inhibit action potential firing (which is only transient), SST also exerts a more sustained inhibitory effect on the exocytotic release of glucagon (39). The amount of glucagon actually being secreted will reflect the combination of the effects on electrical activity and exocytosis. The mathematic model used here provides a powerful tool to investigate the cause of dysregulation of glucagon secretion seen in diabetes.
